# Mothers’ beliefs and barriers about childhood diarrhea and its management in Morang district, Nepal

**DOI:** 10.1186/1756-0500-5-576

**Published:** 2012-10-24

**Authors:** Mukhtar Ansari, Mohamed Izham Mohamed Ibrahim, Mohamed Azmi Hassali, P Ravi Shankar, Arun Koirala, Noor Jang Thapa

**Affiliations:** 1 Discipline of Social and Administrative Pharmacy, School of Pharmaceutical Sciences, Universiti Sains Malaysia, Penang, 11800, Malaysia; 2 College of Pharmacy, Qatar University, Doha, Qatar; 3 Department of Pharmacology, KIST Medical College, Lalitpur, Nepal; 4 Social Action for Rural Health and Development of Nepal (SARHDON), Janapath-tole, Biratnagar, Morang, Nepal; 5 Department of Pharmacology, Nobel Medical College Teaching Hospital, Biratnagar, Nepal

**Keywords:** Barriers, Beliefs, Childhood diarrhea, Management, Mothers, Nepal

## Abstract

**Background:**

In developing countries, mothers usually manage diarrhea at home with the pattern of management depending on perceived disease severity and beliefs. The study was carried out with the objective of determining mothers’ beliefs and barriers about diarrhea and its management.

**Methods:**

Qualitative methods involving two focus group discussions and eight in-depth interviews were used to collect the data. The study was conducted at the following places: Tankisinuwari, Kanchanbari and Pokhariya of Morang district, Nepal during the months of February and March 2010. Purposive sampling method was adopted to recruit twenty mothers based on the inclusion criteria. A semi-structured interview guide was used to conduct the interviews. Written informed consent was obtained from all of the participants before conducting the interviews. The interviews were moderated by the main researcher with the support of an expert observer from Nobel Medical College. The interviews were recorded with the permission of the participants and notes were written by a pre trained note-taker. The recordings were transcribed verbatim. All the transcribed data was categorized and analyzed using thematic content analysis.

**Results:**

Twenty mothers participated in the interviews and most (80%) of them were not educated. About 75% of the mothers had a monthly income of up to 5000 Nepalese rupees (US$ 60.92). Although a majority of mothers believed diarrhea to be due to natural causes, there were also beliefs about supernatural origin of diarrhea. Thin watery diarrhea was considered as the most serious. There was diversity in mothers’ beliefs about foods/fluids and diarrhea management approaches. Similarly, several barriers were noted regarding diarrhea prevention and/or management such as financial weakness, lack of awareness, absence of education, distance from healthcare facilities and senior family members at home. The elderly compelled the mothers to visit traditional healers.

**Conclusions:**

There were varied beliefs among the mothers about the types, causes and severity of diarrhea, classification of foods/fluids and beliefs and barriers about preventing or treating diarrhea.

## Background

Diarrhea is a global problem and is defined as ‘having loose or watery stools at least three times a day or more frequently than normal for an individual’. Rehydration is a cornerstone of treatment of diarrhea to prevent dehydration [1].

People residing in rural and remote areas of developing nations, prefer to manage diarrhea at home [2-4]. Diarrhea is considered to be of both natural as well as supernatural origin [5-7]. Furthermore, it is believed that diarrhea of supernatural origin is not related to hygiene, and there is no role of oral rehydration solution (ORS) and other biomedical approaches unless traditional therapies are adopted [8,9]. In addition to the mothers’ beliefs the beliefs of family members also govern the choice of foods and fluids during diarrhea [10].

In Nepal, the major risk factors in acquiring diarrhea are poor water quality and sanitary conditions, unhygienic stool disposal, contamination of food items and poor household economic conditions [5,11]. Annually, about 30,000 to 45,000 children under five die due to diarrhea in Nepal [12,13]. The decision to seek healthcare is governed by the perceived severity of the disease. However, mothers’ beliefs and traditions act as a barrier to seeking and adopting healthcare [14]. This study aimed to determine mothers’ beliefs and barriers about diarrhea and its management.

## Methods

Qualitative methods involving Focus Group Discussions (FGDs) and In-Depth Interviews (IDIs) were employed by the main researcher to determine mothers’ beliefs and barriers about diarrhea and its management. However, none of the methods employed were free of limitations. Group discussions may not be suitable if the topic is too personal or sensitive to be discussed in a group or there may be the possibility of opinion being modified by listening to the views of other participants. Similarly, in-depth interviews require many interviews for the saturation of data and thus time consuming, accuracy of the data may be an issue and there may be interviewer bias. Therefore, a mixed approach of in-depth interviews (each interview with a single participant) and group discussions was used.

The study was conducted at the following places: Tankisinuwari, Kanchanbari and Pokhariya of Morang district, Nepal during the months of February and March 2010. Morang is a district in the terai (flatlands) of eastern Nepal adjoining India. The study was approved by the Research and Ethics Committee of Nobel Teaching Hospital, Biratnagar, Nepal.

The study participants were recruited from among general duty attending staffs of Nobel Medical College Teaching Hospital representing different ethnicities of Eastern Nepal such as Brahmans/Chhetris (*e.g. Dahal, Ojha, Karkee*), Terai dalits (*e.g. Mehetar/Halkhor, Musahar/Rishidev*), Terai other castes (*e.g. Sharma/Badhai, Teli, Mandal*) and Adivasis/Janjatis (*e.g. Newar, Rajbanshi, Gurung*). The examples stated in parentheses indicate some of the castes under each of the four ethnicities. Brahmans/Chhetris are mainly engaged in government jobs, politics, and army and are the ruling elite of the nation. Terai dalits (the untouchables) are among the most deprived social groups of Nepal and their traditional occupations are cleaning/sweeping, rat catching and working as agricultural labors. Terai other castes constitute people of different castes involved in various occupations such as carpentry, farming and working on daily wages. Adivasis/Janjatis also comprise people from different castes having various traditional occupations [15].

The participants of FGDs and IDIs were different and separate, and none of the participants in group discussions was over pressurized on ‘must say’ basis. The participants were eligible to be enrolled in the study if they were mothers between the age of 25-30 years and had child/children (under the age of 45 months) with diarrhea at the time of study or in the preceding 3 to 6 months, could understand and speak Nepali well and were willing to participate in the study. The mothers of age group 25-30 years were selected because they are supposed to have some prior experiences of taking care of their children and could actively participate in the interviews/discussions.

A total of two FGDs and eight IDIs involving 20 participants were conducted. Each of the FGDs consisted of six participants from different ethnic groups as stated earlier. As the purpose of this study was to obtain more information on beliefs and barriers using limited number of interviews, so a purposeful sampling method was adopted to select the participants from different ethnic groups.

The instrument used in this study was a semi-structured interview guide developed on the basis of the research objectives. For face and content validity, the instrument was pretested among eight participants and necessary amendments such as modifying the confusing words and even certain questions, and the addition of some probing words were made in the instrument according to the results of the pretesting and the comments from three experts. The research experts were from Nobel Medical College and Social Action for Rural Health and Development of Nepal (SARHDON), Biratnagar, Nepal. The expert from Nobel Medical College was directly involved in the interviews as an observer, whereas the remaining two experts from SARHDON were consulted to comment on the findings of the pilot study. It is difficult to maintain reliability of an open ended interview due to lack of exact replication of the interview process. However, reliability of the instrument was improved by clearly setting out the research process, strictly sticking to the content of the guide question, use of the same interviewer for all the interviews and cross checking the data. The final version of the instrument was in the Nepali language and all of the participants were able to understand and speak Nepali regardless of their ethnicity. Written informed consent was obtained from the participants before conducting the interviews. In FGDs, the seating arrangement of the participants was U shaped and the discussions were carried out at a place where there would be no interruptions. Participants were numbered from one to six with a tag card and number. All the participants were instructed to participate actively in the discussions without any hesitation and to mention their tag number each time before they put forward their views. Furthermore, it was highlighted that none of the participants should comment on others’ views as wrong or right, but only expresses their own views. Apart from this, less active participants were encouraged by the moderator from time to time to express their views. The discussions were moderated by the researcher under the observation and support of an expert observer with a qualification of Master in Public Health. The interviews were recorded with the permission of the participants and notes were written by a trained note-taker. The same interviewer, observer and note-taker were involved in all of the IDIs, in which the interviews were face to face and in depth rather than in a group. Each of the FGDs and IDIs lasted approximately for about 80 and 20 minutes respectively.

Complete transcripts of the interviews were prepared and analyzed, together with the recordings of the interviews. The analyzed data were categorized into different themes which were verified by colleagues and experts, and finally the responses were translated into English. Within method triangulation was worked out to gather themes from the interviews and thematic content analysis (TCA) was conducted [16]. Within method triangulation is a type of methodological triangulation in which the findings of two or more techniques of the same method such as qualitative method, e.g. FGDs and IDIs are executed to enhance confidence in the ensuing findings. The common responses were enumerated in the results section. Quotations were used to illustrate the key emergent themes about diarrhea and its management. The quotes were identified by the nature of interview (e.g. FGD or IDI) followed by the participants.

## Results

Twenty mothers participated in the interviews and most (80%) of them had no education. Table 1 shows the demography of the participants. It was not feasible to tabulate each of the responses. Thus, only some key emergent themes have been communicated in Table 2.

**Table 1:** Demographic characteristics of the participants (n = 20)

**Characteristics**		**Number**	**Percentage**
Mothers’ age	25-26 years	9	45
	27-28 years	6	30
	29-30 years	5	25
Mothers’ education	No education	16	80
	Primary education	3	15
	Secondary education	1	5
Family monthly income	Up to 5000	15	75
(NRs) (1 US$ = 82.08 NRs)	5001-10,000	5	25
Ethnicity	Terai/Madhesi dalit castes^1^	7	35
	Terai/Madhesi other castes^2^	3	15
	Adivasi/Janjati^3^	5	25
	Brahman/Chhetri^4^	4	20
	Others^5^	1	5

Examples of the castes of mothers under each ethnicity: 1-Mehetar/Halkhor, Rishidev/Musahar; 2-Sharma/Badhai, Sah/Teli, Mandal; 3-Newar, Rajbanshi, Gurung; 4-Dahal, Ojha, Karkee; 5-Others.

**Table 2:** Mothers’ beliefs about diarrhea and its management (n = 20)

**Themes**	**Examples**	**Number**	**Percentage**
Causes of diarrhea*	Dirt and lack of cleanliness	18	90
	Disposal of stool near home in open air	6	30
	Change in weather	3	15
	Carelessness of mother	2	10
	Traditional/supernatural	3	15
The most dangerous diarrhea	Thin watery stool	16	80
	Red stool	3	15
	Green stool	1	5
Management*	Oral rehydration solution	15	75
	Salt sugar water solution	12	60
	Traditional	3	15
Beneficial foods/fluids*	Home available foods/fluids	17	85
	Mother’s breast milk	12	60
	Green leafy vegetables	6	30
	Potato boiled in cow’s milk	3	15
Harmful foods/fluids*	Spicy and oily foods/fluids	19	95
	Stale/rotten foods/fluids	16	80
	Hard foods/fluids	8	40
	Cold foods/fluids	4	20
Beliefs toward prevention or management of diarrhea*	Modern biomedical	18	90
	Traditional	1	5
	Modern plus traditional	3	15
Barriers toward prevention or management of diarrhea*	Financial weakness	15	75
	Lack of awareness	16	80
	Lack of place for defecation	9	45
	Distance of water source	6	30
	Distance of healthcare facilities	8	40

* The participants may have one or more views, thus the aggregate percentage may be more than 100%.

### Perceived types of diarrhea

A total of nine different types of diarrhea were identified from the interviews such as thin watery stool (*paani jasto paatlo disha*), red stool (*raato disha*), green stool (*hariyo disha*), stool like sputum (*singaan jasto disha*), oily stool (*tel jasto disha*), black stool (*kaalo disha*), stool like egg yolk (*andaa ko girkha jasto disha*), yellow stool (*pahelo disha*) and stool like mustard flower (*tori ko ful jasto disha*). When mothers were asked about the most serious diarrhea, they mostly opined thin watery diarrhea, but I think: *“Green diarrhea is the most dangerous because it happens due to cold and I have seen a child who died due to this type of diarrhea” (FGD-mother 1).*

### Causes of diarrhea

The most common causes of diarrhea were dirt and lack of cleanliness. However, *“Witchcraft (boksi/daaen) and teething are leading causes of diarrhea in villages” (FGD-mother 2 and 3)*.

### Management of diarrhea

Home available foods/fluids, e.g. loose soft rice and pulse/cereal water, oral rehydration solution, salt sugar water solution and more frequent breast feeding were the most favored approaches for the management of diarrhea. However, it was believed that all types of diarrhea are not manageable with ORS and salt sugar water (SSW) solutions. Certain diarrheas require adopting traditional/superstitious methods like exorcism (*dhami-jhankri*). Dhami-Jhankri is a Nepali traditional healer who uses incantation (*tantra-mantra*) to treat the diseases: *“There is no role of ORS and enough water in diarrhea due to teething. I applied ash of dried animal dung with finger twice daily at the site of teething for about one week and it was cured” (FGD-mother 1).* I think *“For green diarrhea which is due to cold, the massage to the whole body of the child with caraway seeds or garlic boiled in edible oil would be helpful for quick recovery. But, red and watery diarrheas require immediate hospitalization” (IDI-mother1).*

### Beneficial foods/ fluids

Mothers were mostly in favor of home available foods and fluids. However, there were contradictory opinions among the mothers about whether cow milk, yogurt, fish and meat are harmful or beneficial during diarrhea: *“Only a special type of ripened banana (maalbhog kera) is beneficial during diarrhea but not any other type, as they further aggravate the diarrhea’ (FGD-mother 4).*

### Harmful foods/fluids

Spicy, oily and rotten food items were commonly believed to be harmful during diarrhea. Generally mothers’ breast milk can be given during diarrhea but: *“Some mothers’ milk are harmful by nature and it should be stopped during diarrhea” (FGD-mother 5).*

### Chances of getting diarrhea and its consequences

Lack of cleanliness, harmful foods/fluids, unhygienic behaviors and lack of proper child care were believed to be strongly associated with episodes of diarrhea. Almost all the participants agreed on the serious consequences of diarrhea. It was further added that parents may have to bear the stress; child may become weak and even die if diarrhea is not treated in time.

### Beliefs about preventing or treating diarrhea

There were both modern and traditional beliefs among the mothers about preventing or treating diarrhea. Although ORS is the cornerstone for the management of diarrhea, all types of diarrhea were not managed with ORS: *“I believe in herbs but not in traditional healers. However, my mother took my baby to dhami-jhankri, a traditional healer and she was cured. If hospital is far, we should sometimes visit traditional healers for the sake of child” (IDI-mother 2). “We can give plenty of water to replace the water lost from the body, but we should stop giving fluids such as soups and ORS as they increase the diarrhea” (IDI-mother 3).*

### Barriers about preventing or treating diarrhea

Barriers of varied nature such as financial weakness, lack of awareness, lack of education, absence of place for defecation, behavior, distance of water source and healthcare facility emerged from the interviews: *“Influence of traditions and the presence of elderly at home are the most important barriers toward adopting modern actions in treating diarrhea. Mother in law pressurizes us to treat our child at home and to visit traditional healers” (FGD-mother 6). “Instability in the country is also a problem in getting proper healthcare. I spent about 24 hours on the road due to a sudden strike on the way to hospital” (IDI-mother 2).*

## Discussion

This study was conducted with the aim of determining mothers’ beliefs about various types of diarrhea and its severity, perceived causes, management approaches (traditional and modern), classifying foods/fluids during diarrhea and the barriers toward adopting modern biomedical approaches in preventing or treating diarrhea. Diarrhea is a worldwide problem and there may be diverse beliefs and practices among the mothers about diarrhea across the globe. Several factors might contribute to beliefs and practices which may vary according to region, country, ethnicity, culture and geographical location.

There were varying beliefs about diarrhea and its management among the mothers of different ethnicities/castes (*e.g. Mehetar, Gurung, Chhetri, Bahun, Sah/teli, Rajbanshi and Newar*) of eastern Nepal. Diversity in beliefs (e.g. traditional) among the mothers can be a major obstacle toward adopting modern biomedical approaches.

This study identified various factors (e.g. natural as well as supernatural) as the causes of diarrhea which is in accordance with the findings of the study conducted in rural setting of Kenya [7]. Majority (80%) of the mothers believed thin watery diarrhea as the most dangerous, hence mothers may be more attentive to this type of diarrhea and may neglect or pay less attention toward the diarrhea of other types [2,17,18]. The beneficial role of green banana in diarrhea is well known, but the finding of this study pinpointed ripened *maalbog kera*, a special type of banana in eastern Nepal as beneficial during diarrhea [19]. This may affect the management of diarrhea at home due to reluctance to use available bananas of other types. Depending on the causes and types of diarrhea as perceived by the mothers, their priorities and treatment patterns may also vary as evident from the findings. There were both modern and traditional beliefs and practices among mothers about diarrhea management.

Most of the mothers who prefer to adopt modern methods of treating diarrhea may not be able to do so due to barriers. Although all the barriers cannot be modified or overcome, a properly designed educational intervention can be a suitable approach to modify some of the barriers (e.g. lack of awareness) and even the traditional beliefs (e.g. beliefs in exorcism).

There were certain limitations of the study such as the study was restricted only to three locations of eastern Nepal, the numbers of interviews were limited and the study findings cannot be generalized as purposive sampling method was adopted to enroll the participants.

## Conclusions

There were varying beliefs of natural as well as supernatural origin among the mothers about the types of diarrhea, its severity, causes and management, beneficial and harmful foods/fluids, efficacy of the advised modern health actions, and beliefs and barriers about preventing or managing diarrhea.

The authors would like to recommend future research on beliefs, traditions and barriers in a larger population and at different geographical locations in the country. Besides considering the beliefs or attitude of mothers, there is a need for taking into account the beliefs or attitude of senior members of the family (e.g. husband and mother/father in law) as they may play a vital role in deciding whether to seek healthcare or not for an illness (e.g. diarrhea) and if yes, whether to seek traditional or modern biomedical help. The study findings may help the future researchers and policy makers to incorporate the findings while conducting studies at wider level.

## Competing interests

All authors declared that they have no competing interest.

## Authors’ contributions

MA designed the study, analyzed and interpreted the data and drafted the manuscript. MIMI participated in designing the instrument and interpretation of the data and editing the manuscript. MAH participated in designing the instrument and interpretation of the data and editing the manuscript. PRS was involved in interpretation of the data and editing the manuscript. AK participated in translation of data and identification of themes and interpretation of data. NJT participated in translation of data and identification of themes. All authors read and approved the final manuscript.
